# Epilepsia Open—December 2025 Announcements

**DOI:** 10.1002/epi4.70198

**Published:** 2025-12-19

## ILAE CONGRESSES

**15th ILAE School on Pre‐Surgical Evaluation for Epilepsy and Epilepsy Surgery**

19–23 January 2026

Brno, Czech Republic

**19th Latin American Summer School on Epilepsy**

23–28 February 2026

Guadalajara, Mexico

**8th ILAE School on EEG in the First Year of Life**

30 March–2 April 2026

Cambridge, UK

**5th International Summer School of Neuropsychology**

19–24 April 2026

Picardy, France

**ILAE School on Neuroimaging 2026**

6–9 May 2026

Potsdam, Germany

**XIV Congreso Latinoamericano de Epilepsia**

16–18 May 2026

Lima, Peru

**1º Curso Latinoamericano de EEG Pediátrico**

6–9 August 2026

TBC

**16th European Epilepsy Congress**

5–9 September 2026

Athens, Greece

**16th International Summer School for Neuropathology and Epilepsy Surgery**

10–13 September 2026

Erlangen, Germany

**16th Asian & Oceanian Epilepsy Congress**

19–22 November 2026

Kuala Lumpur, Malaysia

## 2027

**37th International Epilepsy Congress**

28 August–1 September 2027

Amsterdam, Netherlands

## ILAE WEBINARS

**YES Education Series: Management of Acute Repetitive Seizures & Status Epilepticus**

11 December 2025

**ILAE e‐Forum: EEG: When, why and how?**

12 December 2025

**Side Effects of Anti‐Seizure Medications**

12 December 2025

**Genetic des encéphalopathies développementales et épileptiques**

16 December 2025

**EEG and Coma**

20 January 2026

## OTHER CONGRESSES

**Encephalitis 2025**

3–4 December 2025

London, UK & Online

**AES 2025 Annual Meeting**

5–9 December 2025

Atlanta, Georgia, USA

**British Neurosurgical Research Group Meeting 2025**

8–9 December 2025

London, England

**4th Advanced Imaging in Epilepsy**

12–14 December 2025

Rome, Italy

**Paediatric Epilepsy Training 1**

13 December 2025

Tangier, Morocco

## 2026

**Paediatric Epilepsy Training 1 – Virtual**

10 February 2026

Online

**4th European Congress of Neurology and Neuropsychiatry**

23–24 February 2026

Paris, France

**Paediatric Epilepsy Training 2 – Bristol**

26–27 February 2026

Bristol, UK

**Paediatric Epilepsy Training 3 – Bristol**

26–27 February 2026

Bristol, UK

**4th International Artificial Intelligence in Epilepsy and Neurological Disorders Conference**

16–19 March 2026

San Juan, Puerto Rico

**EPIPED Course: Treatment Strategies in Pediatric Epilepsies**

22–25 April 2026

Girona, Spain

**18th Eilat Conference on New Antiepileptic Drugs and Devices**

3–6 May 2026

Madrid, Spain

**20th Specialist Epilepsy Teaching Weekend**

16–17 May 2026

Birmingham, UK

**64th Annual Meeting of the German Society for Epileptology**

10–13 June 2026

Würzburg, Germany

**Paediatric Epilepsy Training 2 – Leeds**

11–12 June 2026

Leeds, UK

**Paediatric Epilepsy Training 3 – Leeds**

11–12 June 2026

Leeds, UK

**22nd San Servolo Advanced Epilepsy Course**

20–31 July 2026

San Servolo (Venice), Italy

**Epilepsy Imaging in Resource Strained Settings**

31 August – 12 September 2026

TBC

**1st Swiss Neuro Week**

18–20 November 2026

Bern, Switzerland

